# Porous silicon/Ni composites of high coercivity due to magnetic field-assisted etching

**DOI:** 10.1186/1556-276X-7-384

**Published:** 2012-07-11

**Authors:** Petra Granitzer, Klemens Rumpf, Toshiyuki Ohta, Nobuyoshi Koshida, Peter Poelt, Michael Reissner

**Affiliations:** 1 Institute of Physics, Karl Franzens University Graz, Universitaetsplatz 5, Graz, A-8010, Austria; 2 Graduate School of Engineering, Tokyo University of Agriculture and Technology, 2-24-16 Nakacho, Koganei, Tokyo, 184-8588, Japan; 3 Institute for Electron Microscopy, University of Technology Graz, Steyrergasse 17, Graz, A-8010, Austria; 4 Institute of Solid State Physics, Vienna University of Technology, Wiedner Hauptstr. 8, Vienna, A1040, Austria

**Keywords:** Magnetic field-assisted anodization, Porous silicon, Magnetic nanostructures, 68.65.-k, 75.75.-c, 81.07.-b

## Abstract

Ferromagnetic nanostructures have been electrodeposited within the pores of porous silicon templates with average pore diameters between 25 and 60 nm. In this diameter regime, the pore formation in general is accompanied by dendritic growth resulting in rough pore walls, which involves metal deposits also offering a branched structure. These side branches influence the magnetic properties of the composite system not only due to modified and peculiar stray fields but also because of a reduced interpore spacing by the approaching of adjacent side pores. To improve the morphology of the porous silicon structures, a magnetic field up to 8 T has been applied during the formation process. The magnetic field etching results in smaller pore diameters with less dendritic side pores. Deposition of a ferromagnetic metal within these templates leads to less branched nanostructures and, thus, to an enhancement of the coercivity of the system and also to a significantly increased magnetic anisotropy. So magnetic field-assisted etching is an appropriate tool to improve the structure of the template concerning the decrease of the dendritic pore growth and to advance the magnetic properties of the composite material.

## Background

In recent years, the demand on nanostructured materials increased enormously not only because of the miniaturization of devices but also because of the appearance of modified physical properties of nanosized objects compared to their bulk material. Nanostructures can be achieved by a top-down or bottom-up approach, whereas nanostructuring by self-organization is an emerging topic due to less costs, less time consumption and mostly a simple fabrication procedure.

Porous silicon can be formed with various morphologies [1] exhibiting structures between a few nanometers and several tens of micrometers, and in its different natures, it is a versatile material for a broad range of applications in optics [2], acoustics [3] and sensor technology [4], and in biomedicine, it is promising due to its biodegradability and biocompatibility [5]. The possibility of achieving straight pores renders porous silicon a good template material for the deposition of various metals [6], whereas the case of merging ferromagnetic nanostructures with a semiconducting substrate results in a hybrid material, which offers the electronic properties of silicon and the nanomagnetic behavior of the precipitates. The deposition of the metals within the pores can be achieved electroless, electrochemically or by vapor transport methods such as atomic layer deposition or a form of chemical vapor deposition.

The fabrication of specimens with specific magnetic properties can be reached due to the possibility of modifying the geometry and the packing density of the precipitated metal nanostructures as well as of varying the morphology of the template [7]. Considering the deposition of metal wires within the pores, a magnetic anisotropy between the two magnetization directions, with an applied field perpendicular (easy axis) and parallel (hard axis) to the surface, can be observed. The metal wires interact dipolarly not only within the pores but also between adjacent pores, depending on the pore distance. To enhance this anisotropy, the magnetostatic interactions between adjacent pores have to be decreased. Thus, the application of a magnetic field during the etching procedure has been employed to achieve smoother pore walls, leading also to smoother metal deposits, which results in a bigger effective distance between adjacent metal structures.

## Methods

**Figure 1:**
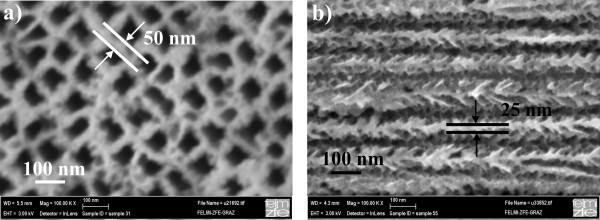
**SEM images of a porous silicon specimen.** ( **a**) Top-view micrograph showing pores with an average diameter of 60 nm and a mean distance between the pores of 50 nm. ( **b**) The cross-sectional image demonstrates that the effective mean pore distance is less than 50 nm due to the occurring side branches.

The conventionally etched porous silicon samples were prepared by anodization of highly n-doped silicon (10^19^/cm^3^) in aqueous/ethanoic acid solution at room temperature [8]. To achieve average pore diameters of 60 nm, a current density of 100 mA/cm^2^ has been applied. In this case, the pore formation is accompanied by the growth of small side pores exhibiting a length smaller than half of the pore distance, which ensures that the pores in general are not interconnected. Looking at a top-view scanning electron microscopy (SEM) image of a porous silicon specimen (Figure 1a), the mean pore distance is 50 nm, whereas the corresponding cross-sectional image shows that the effective mean distance between the pores is smaller (around 25 nm) due to the occurring side pores (Figure 1b).

**Figure 2:**
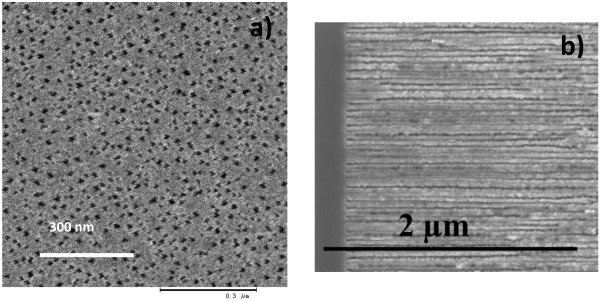
**Magnetic field-assisted anodized specimen.** ( **a**) Top-view SEM image of a magnetic field-assisted anodized specimen showing pores with an average diameter of 25 nm. ( **b**) Corresponding image of the cross-sectional region showing the less dendritic growth of the pores.

To improve the porous structure, which means to diminish the dendritic growth, a magnetic field of 8 T has been applied perpendicular to the sample surface during the anodization process. Furthermore, the temperature of the electrolyte has been kept at 0 °C. The resulting morphology exhibits less and shorter side pores and a reduced pore diameter of about 25 nm (Figure 2).

Both types of templates, conventionally etched and magnetic field-etched ones, have been filled with Ni electrochemically. As electrolyte, a solution consisting of NiCl_2_, NiSO_4_ and H_3_BO_3_ has been employed. The galvanic deposition has been performed in a pulsed way [9]. In choosing a current density of 12 mA/cm^2^ and a pulse frequency of 0.2 Hz, Ni-nanowires of a few micrometer in length were successfully embedded into the pores. The deposited Ni structures exhibit in all cases a wire-like structure with an aspect ratio up to 100. The precipitated Ni-wires within conventionally etched porous silicon offer roughness with a branched structure. In using the magnetic field-etched porous silicon as a template, the precipitated Ni-wires are smoother, and the mean distance between neighboring pores is increased up to 50 nm.

To investigate the prepared nanocomposite systems structurally, mainly SEM and EDX spectroscopy is used. Magnetic characterization has been carried out by SQUID magnetometry and a vibrating sample magnetometer.

## Results and discussion

Considering the dissolution process of silicon, holes diffusing to the silicon/electrolyte interface attack the silicon atoms, but the motion of the holes cannot be controlled easily. The application of a magnetic field perpendicular to the sample surface during the anodization process controls this motion of the holes moving from the bulk silicon to the pore-tip region. In this case, the holes only reach the pore tips and do not diffuse any further to the pore walls. Holes moving in the growth direction of the pores contribute to the formation process, whereas holes with a direction that deviates from the growth direction feel the Lorentz force, which is induced by the applied magnetic field and do not contribute to the dissolution of the silicon. This experimental arrangement results in smaller pore diameters and smoother pore walls compared to conventional etching with adequate electrochemical parameters [10].

Electrodeposition of Ni within these less dendritic templates results also in smoother Ni-wires with less side pores. Thus, on the one hand, the geometry of the wires with less dendrites and, on the other hand, the resulting bigger distance between adjacent pores influence the magnetic properties of the composite system. Comparing the samples with deposited Ni-wires, in which the porous silicon was prepared by either conventional etching or by magnetic field-assisted etching, a significant modification of the magnetic properties was observed.

The magnetic behavior of samples anodized without magnetic field exhibits coercivities of *H*_C_ = 270 Oe (measured at *T* = 4 K) and 160 Oe (measured at *T* = 250 K), whereas magnetic field-assisted anodized samples offer a more-than-doubled coercivity, *H*_C_ = 660 Oe (measured at *T* = 4 K) and *H*_C_ = 540 Oe (measured at *T* = 250 K) due to the reduction of magnetostatic coupling between adjacent pores, which increases the magnetization reversal field of the individual nanowire. Some values are summarized in Table 1. The achieved coercivity is still far less than the theoretically obtained value of the coercivity of a monocrystalline individual nanowire with *H*_C_ = 3,400 Oe. Reasons for the reduction of the coercivity *H*_C_ are the still-occurring weak dipolar coupling between Ni-wires of adjacent pores, an imperfect cylindrical shape of the nanowires (due to the roughness of the pore walls) and, especially, the end of the wires showing an arbitrary geometry (not flat), which strongly influences the stray fields of the Ni-wires [11]. Further enhancement of the coercivity to come closer to the theoretical value of a single wire could be reached by improving the nanowire growth (cylindrical shape with less roughness and monocrystallinity) and further decreasing the magnetostatic interactions between wires by increasing the distance between the pores.

**Table 1:** **Magnetization data measured at*T***  **= 4 K and*T***  **= 250 K**

	**Coercivity (Oe) magnetic field perpendicular to surface**	**Coercivity (Oe) magnetic field parallel to surface**	**Remanence M/M**_**S**_**(emu) magnetic field perpendicular to surface**	**Remanence M/M**_**S**_**(emu) magnetic field parallel to surface**
*T =* 4 K (conv.)	270	180	0.42	0.36
*T* = 100 K (conv.)	200	110	0.40	0.28
*T* = 250 K (conv.)	160	75	0.38	0.22
*T* = 4 K (mag.)	660	190	0.85	0.38
*T* = 100 K (mag.)	570	125	0.81	0.34
*T* = 250 K (mag.)	540	100	0.78	0.28

conv., samples prepared by conventional etching; mag., samples prepared by magnetic field-assisted etching.

## Conclusions

In the presented work, the modification of the magnetic properties of porous silicon/Ni nanocomposites caused by magnetic field-assisted pore formation is discussed. The high-aspect-ratio pores fabricated by anodization with an applied magnetic field offer a pore diameter smaller than 30 nm and a remarkable decreased growth of side pores. The deposition of Ni structures within these pores results in a more-than-doubled coercivity (increase from 270 to 650 Oe) and a doubled remanence (from 0.42 to 0.85 emu) of the nanocomposite compared to samples etched without magnetic field, whereas the aspect ratio of the deposited wires is comparable. The enhanced values can be attributed to the reduced magnetostatic interactions between Ni-wires deposited within neighboring pores, mainly caused by smoothened walls of the wires and an enhanced distance between the pores.

## Competing interests

The authors declare that they have no competing interests.

## Authors' contributions

PG performed the sample preparation by conventional anodization as well as the Ni-deposition into all templates. PG and KR carried out the magnetization measurements by SQUID and VSM. TO and NK performed the magnetic field-assisted anodization. PP carried out the SEM investigations. MR assisted in performing the VSM measurements.
